# NF-κB-mediated miR-124 suppresses metastasis of non-small-cell lung cancer by targeting MYO10

**DOI:** 10.18632/oncotarget.3135

**Published:** 2015-01-29

**Authors:** Yingjia Sun, Xinghao Ai, Shengping Shen, Shun Lu

**Affiliations:** ^1^ Lung Tumor Clinical Medical Center, Shanghai Chest Hospital, Shanghai Jiao Tong University, Shanghai 200030, China

**Keywords:** NSCLC, miR-124, MYO10, NF-κB, metastasis

## Abstract

Recently, dysregulation of microRNAs plays a critical role in cancer metastasis. Here, an *in vivo* selection approach was used to generate highly aggressive NSCLC sub-cell lines followed by comparing the microRNAs expression using microarrays. miR-124 was notably deregulated in both highly invasive sub-cell lines and node-positive NSCLC specimens. Over-expression of miR-124 robustly attenuated migration and metastatic ability of the aggressive cells. MYO10 was subsequently identified as a novel functional downstream target of miR-124, and was up-regulated in node-positive NSCLC tissues. Knockdown of MYO10 inhibited cell migration, whereas forced MYO10 expression markedly rescued miR-124-mediated suppression of cell metastasis. Additionally, we found an activated NF-κB-centered inflammatory loop in the highly aggressive cells leading to down-regulation of miR-124. These results suggest that NF-κB-regulated miR-124 targets MYO10, inhibits cell invasion and metastasis, and is down-regulated in node-positive NSCLC.

## INTRODUCTION

As the major problem in the management, metastasis frequently occurs in non-small cell lung cancer (NSCLC), with it being the most critical concern [1–2]. It has been observed that approximately 90% patients die from metastasis rather than primary tumors [3]. And the failure of treatment mainly due to development of a high metastatic potential in NSCLC [4]. Therefore, identification of predictive molecular factors and understanding of the underlying molecular mechanisms during tumor metastasis have major importance and might propose effective therapeutic strategy against NSCLC.

MicroRNAs (miRNAs) are a class of approximately 22-nucleotide non-coding RNA molecules, and have been identified as key negative regulators of gene expression through the endogenous RNA interference machinery [5–6]. Accumulating evidence suggests that miRNAs play a role in diverse biological processes, including proliferation, apoptosis and tumorigenesis [7–8]. Recent studies have also shown that dysregulation of miRNAs is implicated in invasion and metastasis in several human cancer types [9–10]. Although a few miRNAs have been associated with NSCLC metastasis, including miR-143, miR-135b, miR-31 and miR-193a [11–13], identification of novel predictive and therapeutic target remains a large unmet need.

To explore the miRNA that may be involved in NSCLC metastasis, we established a series of high metastatic cell sub-lines from two weakly invasive cell lines (H522 and H1975) using *in vivo* selection systems. And deregulation of miR-124 was found in the cells with high invasive and metastatic capacity. Recently, it was found that miR-124 functioned as a tumor-suppressive microRNA in several cancers [14–16], and decreased expression of miR-124 may be associated with tumorigenesis and metastasis by regulating its target gene [17–18]. In our work, over-expression of miR-124 achieved a decrease in cellular migration and invasion abilities. Although several targets of miR-124 have been validated in different models, the potential roles and related target genes in NSCLC metastasis remain unclear.

In this study, we observed that miR-124 was suppressed in highly aggressive cell lines and node-positive NSCLC tissues. Forced expression of miR-124 greatly impaired NSCLC cell metastasis *in vitro*. Moreover, MYO10 was identified as a direct and functional target of miR-124. Further analyses showed that an IL6-NF-κB inflammatory loop was activated and can deregulate miR-124 expression. Together, our work proposed a novel mechanism driving NSCLC metastasis by NF-κB-mediated miR-124 suppression leading to increased MYO10 expression, providing novel insights for the prevention of NSCLC metastasis.

## RESULTS

### Isolation of subpopulations of NSCLC cells that have a high metastatic potential

**Figure 1:**
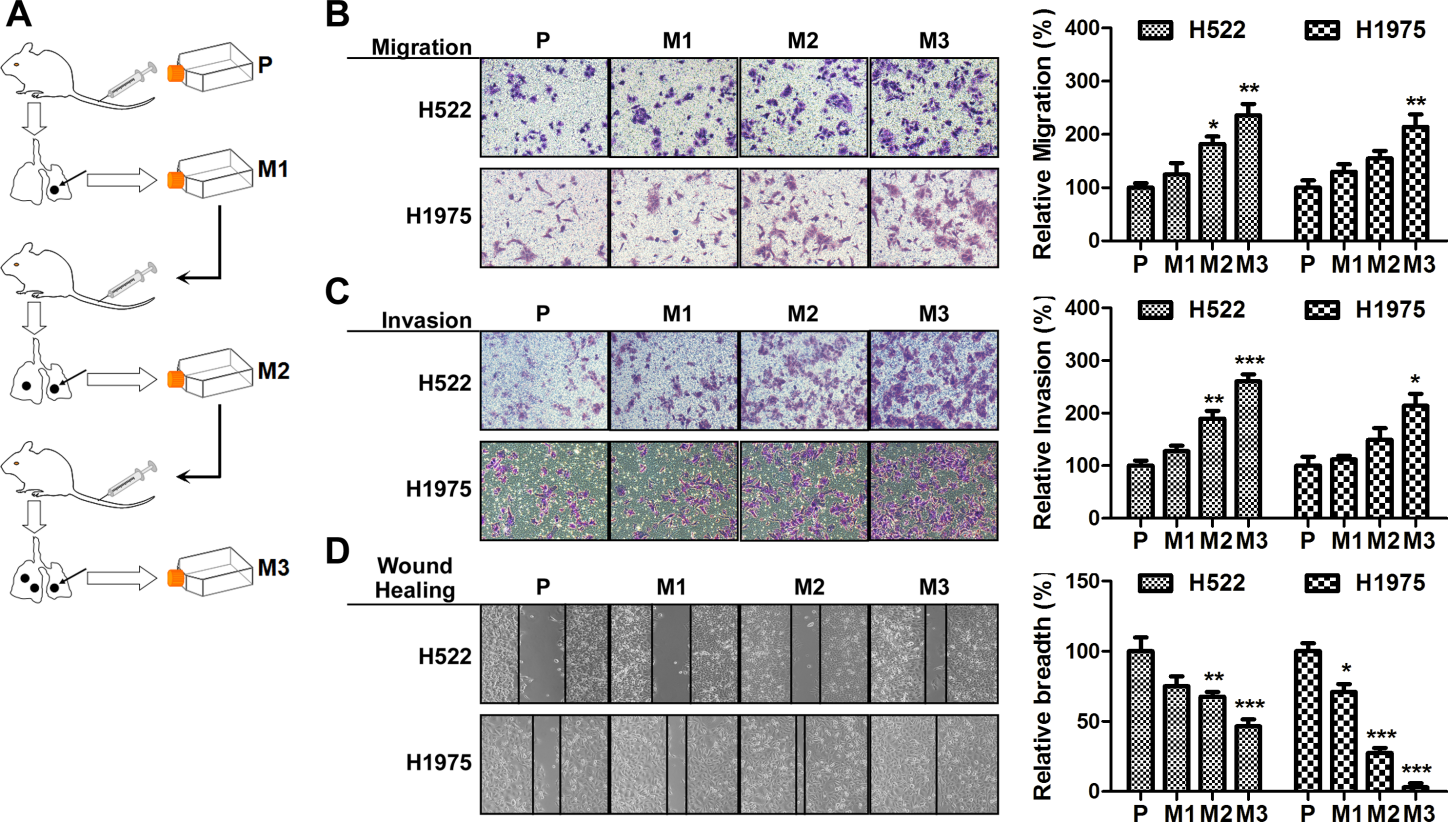
Isolation of subpopulations of NSCLC cells that have a high metastatic potential **(A)** Experimental outline for the generation of highly aggressive NSCLC cell lines. H522 or H1975 cells were intravenously injected into the tail vein of nude mice. After 6 weeks, lung nodules were removed and were expanded *in vitro* and subsequently intravenously re-injected. The high metastatic potential sub-cell lines (after 3 rounds of selection) were compared with the parental cell lines. **(B)** Cell migration assays. Cells were placed into the upper chamber for 24 h. Migrated cells were stained with crystal violet, and counted for quantitative analysis. **(C)** Cell invasion assays. Cells in serum-free medium were seeded into the Matrigel pre-coated upper chamber, and the invading cells were stained and counted for quantitative analysis. **(D)** Wound healing assays. Cell culture plates attached with cells were wounded and cultured for 48 h. The wound healing was determined. Data are presented as mean ± s.e.m.. (*n* = 3). *p < 0.05, **p < 0.01 and ***p < 0.001 for comparing with parental cells.

To identify potential mechanisms of metastasis, we firstly generated a series of highly aggressive NSCLC sub-cell lines using a *in vivo* selection systems. Two NSCLC cell lines, H522 and H1975, were used to form multiple small nodules in athymic nude mice by tail vein injection. The cells were further isolated from tumor nodules followed by a reinjection. After 3 rounds of *in vivo* selection, cells with increased metastatic capacity were obtained as shown in Figure 1A. To evaluate the biological aggressiveness, cell migration and invasion assays were performed. As predicted, we found a progressive increase in the migration ability of cell lines obtained from each rounds (Figure 1B). And the collagen invasion assays showed a similar tendency in cellular invasion (Figure 1C). Wound healing assays demonstrated the established cell lines exhibited improved ability to migrate into the wound (Figure 1D). Among these cells, H522M3 and H1975M3 cells exhibited highly metastatic potential compared with the parental cells, and could be used for further study.

### miR-124 is down-regulated in aggressive NSCLC cells

**Figure 2:**
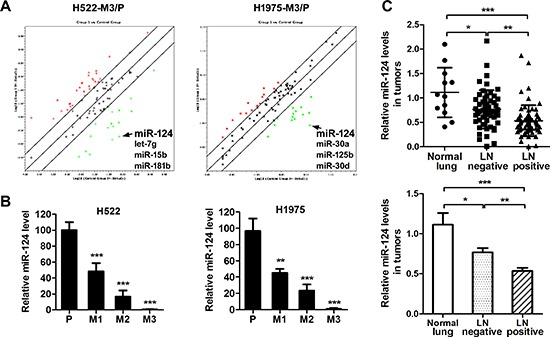
miR-124 is down-regulated in aggressive NSCLC cells **(A)** Analysis of the fold regulation in the microRNAs was performed by a microRNA array. The green points indicate down-regulated miRNAs, and the red points indicate up-regulated miRNAs. **(B)** Relative miR-124 expression in parental and aggressive cells was determined by qRT-PCR. **p < 0.01 and ***p < 0.001 for comparing with parental cells. **(C)** qRT-PCR analysis of miR-124 abundance in human normal lung (*n* = 12) or NSCLC tumors with lymph nodes positive (*n* = 53) or negative (*n* = 65). *p < 0.05, **p < 0.01 and ***p < 0.001.

Since there have only been a few reports on the associations between miRNA gene expression and NSCLC metastasis, we wonder whether miRNAs play a role in induction of a highly aggressive phenotype in our model. The miRNA expression in parental and highly aggressive cells (M3) was examined using miRNA qPCR array, and fold regulation in the miRNAs was analyzed. A panel of miRNAs with significant differential expression between parental and M3 cells was derived through a series of contrasts (Supplementary Table 1), and these miRNAs were selected for further validation and functional characterization. Among these miRNAs, the significant down-regulated expression of miR-124 was consistently observed in the two aggressive sub-cell lines (H522M3 and H1975M3) compared to parental cells (Figure 2A). These results from microarray studies were subsequently validated as demonstrated by qRT-PCR analysis that the miR-124 level was significantly down-regulated in the established aggressive cells compared with parental cells (Figure 2B). Furthermore, miR-124 expression in biopsies from normal lung or patients with lymph node-positive and -negative NSCLC was also examined with qRT-PCR. It was demonstrated that miR-124 was decreased in NSCLC compared with the normal lung tissues, and low miR-124 expression was significantly associated with lymphatic metastasis of the NSCLC (Figure 2C).

### miR-124 inhibits metastases of NSCLC cells

**Figure 3:**
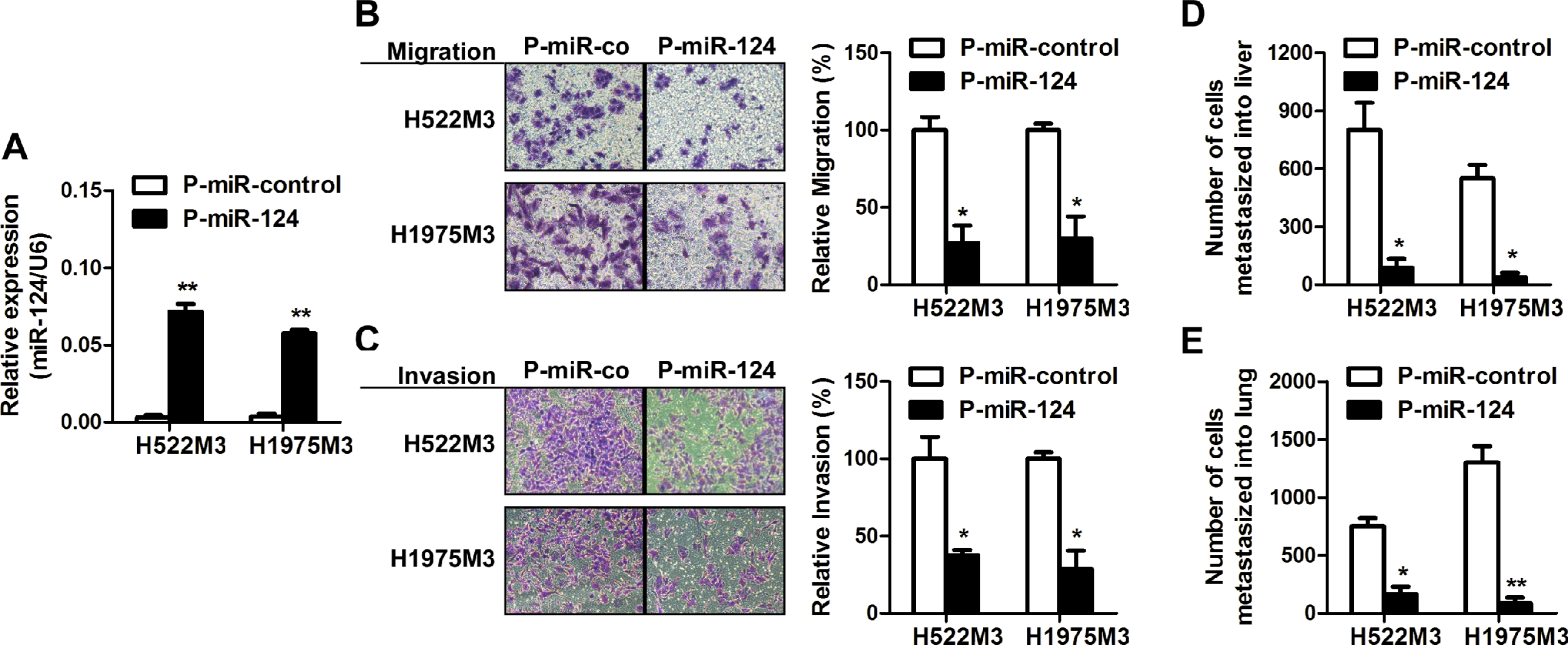
miR-124 inhibits metastases of NSCLC cells **(A)** H522M3 and H1975M3 cells were stably transfected with P-miR-control or P-miR-124, and the expression of miR-124 was examined by qRT-PCR. **p < 0.01. **(B)** Cell migration assays and **(C)** invasion assays examining the effects of miR-124 overexpression on metastatic potentiality of cells. *p < 0.05. **(D)** *In vivo* distant metastasis of cancer cells to livers (D) and lungs **(E)** was examined by a chicken embryo metastasis assay. *p < 0.05 and **p < 0.01.

To imitate the NSCLC cells with constitutive high miR-124 expression, stable transfectant of H522M3 and H1975M3 cells expressing miR-124 was constructed, and the miR-124 level was examined by qRT-PCR (Figure 3A). As shown in Figure 3B and 3C, suppressive role of miR-124 on metastatic potential of NSCLC cells was observed. Highly invasive H522M3 and H1975M3 cells stably expressing miR-124 exhibited impaired capacity of migration and invasion as compared with their counterparts. Moreover, a chicken-embryo metastasis model was used to further validate the role of miR-124 *in vivo* and an ability to affect on distant metastasis. Cells stably transfected with pre-miR-124 (P-miR-124), or scrambled control miRNA (P-miR-control) were inoculated on the top of chorioallantoic membrane (CAM) of ten-day-old chicken embryos. The number of metastasized cells to lung and liver was measured 7 days later. Consistent with the migration and invasion data, miR-124 inhibited metastasis to both liver (Figure 3D) and lung (Figure 3E).

### MYO10 is identified as a functional downstream target of miR-124

**Figure 4:**
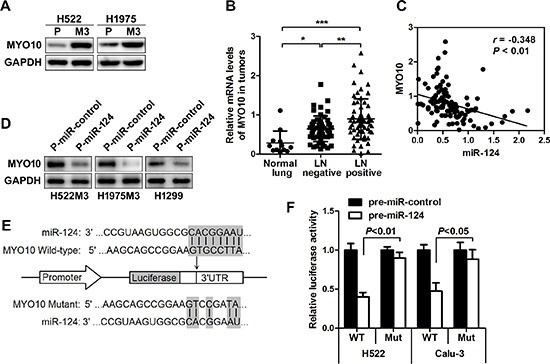
MYO10 is identified as a functional downstream target of miR-124 **(A)** Western blot analysis of the expression of MYO10 in aggressive M3 and parental cells. **(B)** Relative MYO10 expression in human normal lung or NSCLC tumors with lymph nodes positive or negative was determined by qRT-PCR. *p < 0.05. **(C)** The correlation between the expression of MYO10 and miR-124 was analyzed. **(D)** After transfection of P-miR-124, the expression of MYO10 in H522M3, H1975M3 and H1299 cells was examined by Western blot. **(E)** Schematic representation of the human MYO10 3′UTR showing the highly conserved miR-124 binding site, and the pGL3 reporter vectors carrying the wide type or mutated MYO10 3′UTR are indicated. **(F)** The human MYO10 3′UTR vector containing a putative binding site for miR-124 was co-transfected into H522 and Calu-3 cells along with P-miR-124, and the luciferase activity was messured 24 h post-transfection. **p < 0.01.

In order to demonstrate the molecular mechanisms by which miR-124 impairs tumor metastasis in NSCLC, several computational algorithms were used to identify the potential functional targets for miR-124. Using miRecords, an integrated resource for microRNA-target interactions, a panel of molecules was predicted to be potential targets of the miR-124 with eight miRNA target prediction programs (Supplementary Table 2). Expression difference of the predicted potential targets between parental and aggressive cells was further examined. It was identified that MYO10 was commonly up-regulated in the two aggressive cells compared to parental cells (Figure 4A). The increased expression of MYO10 was also observed in NSCLC, particularly in lymph node-positive ones (Figure 4B), and was negatively correlated with miR-124 level in NSCLC tissue biopsies (Figure 4C). This prompted us to study the expression of MYO10 in ectopic miR-124 expressed cells revealing a significant down-regulation (Figure 4D). Additionally, inhibition of miR-124 with anti-miR-124 markedly increased the expression of MYO10, together with the potential of cell migration (Supplementary Figure 1A–1D). Using the miRNA prediction tool, miRanda (v1.9), we identified the phylogenetically conserved miR-124 seed-matching sequences in human MYO10 3′UTR (Figure 4E). Ectopic miR-124 repressed the activity of MYO10 wild-type 3′UTR reporter constructs in dual luciferase reporter assays (Figure 4F), while mutation in miR-124-binding site completely abrogated this repression. Therefore, it was demonstrated that the MYO10 expression could be directly regulated by miR-124 via conserved seed-matching sequences and correlates with the node metastasis of NSCLC disease.

### Re-expression of MYO10 rescues miR-124-mediated suppression of cell aggressiveness

**Figure 5:**
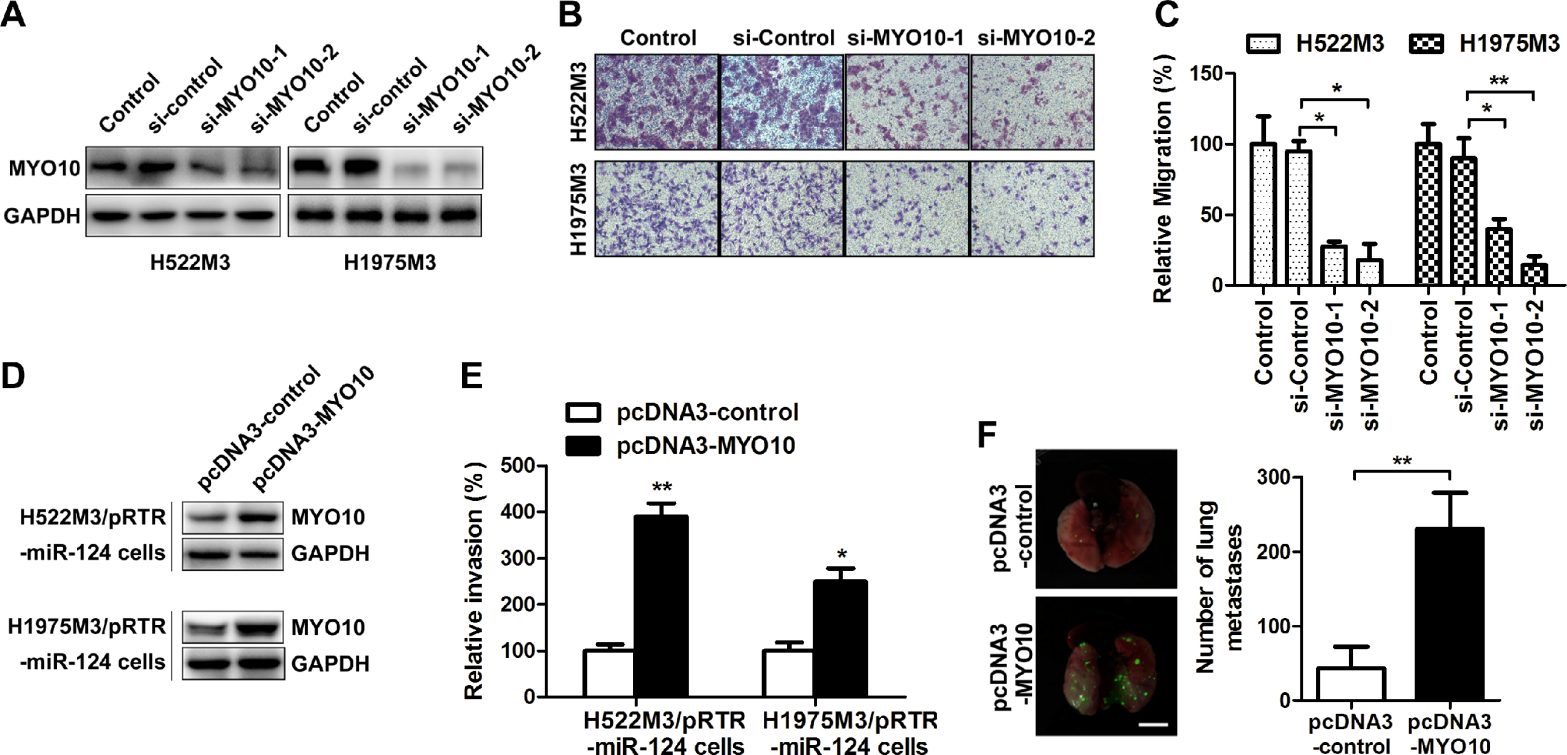
Re-expression of MYO10 rescues miR-124-mediated suppression of cell aggressiveness H522M3 and H1975M3 cells were tansfected with control siRNA (si-control) or MYO10 specific siRNA (si-MYO10), **(A)** the expression of MYO10 was determined by Western blot. **(B)** After transfection, the migratory properties were examined by transwell migration assays. The migrated cells were stained with crystal violet, and **(C)** counted for quantitative analysis. *p < 0.05 and **p < 0.01. **(D)** The expression of MYO10 in the stable transfected cells following re-expression of MYO10 in pRTR-miR-124 cells were analyzed by Western blot. **(E)** After re-expression of MYO10, cell invasion was analyzed by matrigel invasion assays. The invaded cells were summarized in the bar graph. *p < 0.05 and **p < 0.01. **(F)** Representative EGFP imaging revealing metastatic foci on the surface of lungs excised from mice after re-expression of MYO10. Bar, 5 mm. Quantification of EGFP-positive metastatic foci on the surface of the lung. **p < 0.01.

Our findings that elevated MYO10 expression in NSCLC patients was associated with lymph node metastasis led us to hypothesise that MYO10 might be involved with cell motility. MYO10-specific (si-MYO10) and scrambled (si-control) small interfering RNA were used, and the efficacy was examined by Western blot (Figure 5A). As expected, MYO10 depletion with small interfering RNA markedly abrogated cell migration capabilities in aggressive NSCLC cells (Figure 5B and 5C). To further study the roles of MYO10 in miR-124-mediated suppression of aggressiveness in NSCLC cells, we rescued the expression of MYO10 in cells stably expressing miR124 (H522M3/pRTR-miR-124 and H1975M3/pRTR-miR-124 cells) by transfecting the plasmids carrying MYO10 (pcDNA3-MYO10). While cells expressed stably high levels of miR-124 exhibited a decrease in invasion (Figure 3C), re-expression of MYO10 in H522M3/pRTR-miR-124 and H1975M3/pRTR-miR-124 cells, as confirmed by Western blot analysis (Figure 5D), restored the capacity of invasion (Figure 5E). Furthermore, H522M3/pRTR-miR-124 cell containing pcDNA3-control or pcDNA3-MYO10 were injected into the tail vein to investigate MYO10-dependent distant metastasis. The number of metastatic foci on the lung surface was significantly increased after re-expression of MYO10 (Figure 5F).

### An IL6-NF-κB inflammatory loop is activated in aggressive NSCLC cells

**Figure 6:**
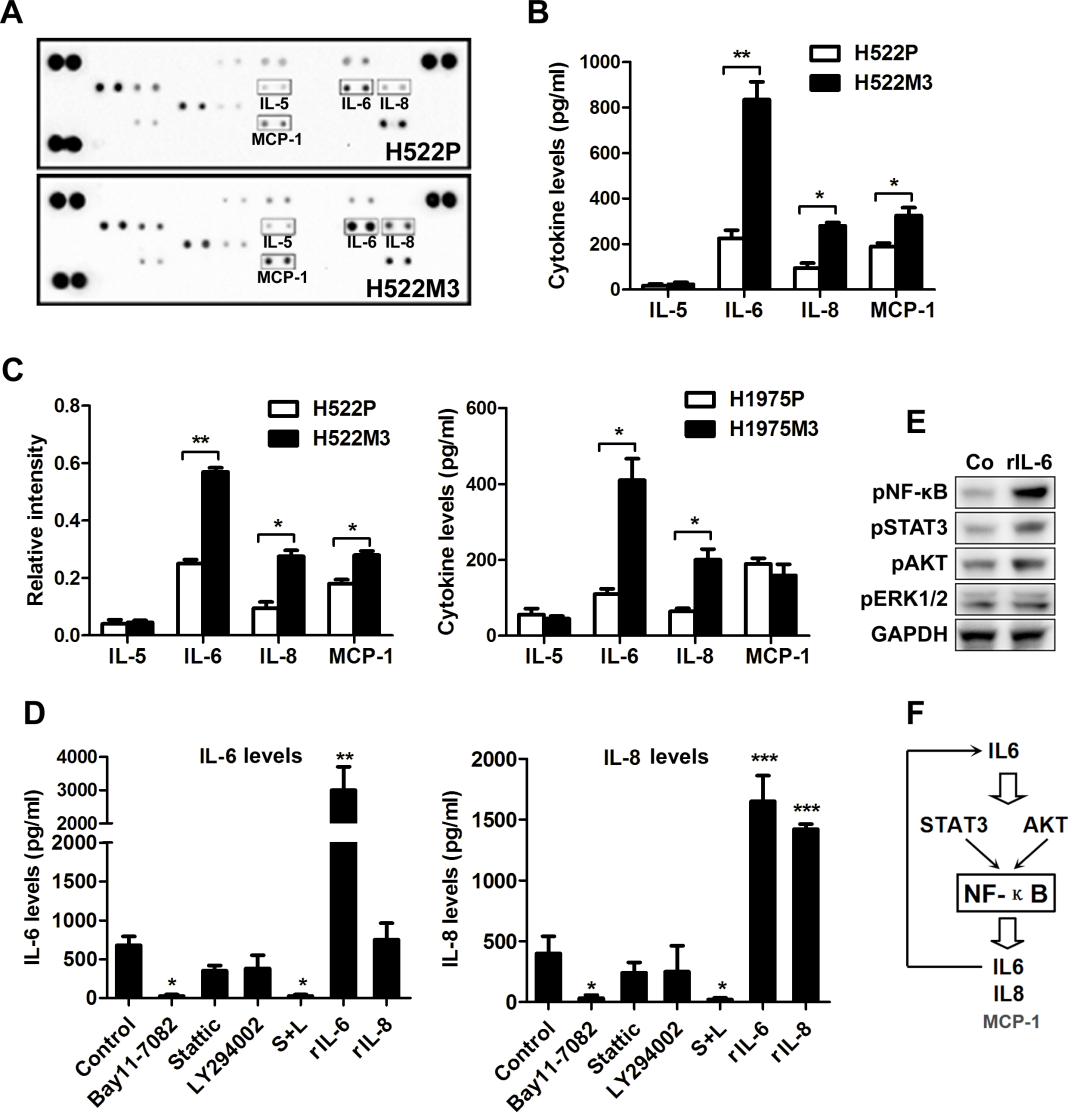
An IL6-NF-κB inflammatory loop is activated in aggressive NSCLC cells **(A)** Cytokine array of conditioned media of parental H522 cells and aggressive H522M3 cells. **(B)** The intensity of each blot compared to control was determined by Kodak image analyzer. **(C)** The secretion of IL-5, IL-6, IL-8 and MCP-1 were examined by ELISA. **(D)** ELISA detecting the secretion of IL-6 and IL-8 in H522 cells after treated with NF-κB inhibitor (10 μM Bay11-7082), or combined inhibition of AKT (10 μM LY294002) and STAT3 (5 μM Stattic), recombinant IL-6 (rIL-6, 50 ng/ml) and recombinant IL-8 (rIL-8, 50 ng/ml). *p < 0.05 and ***p < 0.001. **(E)** Western blot analysis of the expression of pNF-κB, pAKT, pSTAT3 and pERK1/2 in H522 cells after stimulation with rIL-6 (50 ng/ml). **(F)** An IL6-NF-κB feedback loop was schematically illustrated.

Recent studies have demonstrated that a number of cytokines, including IL-6, IL-8 and MCP-1/CCL2, play a role in invasion and metastasis. Utilizing an antibody cytokine array, we examined the levels of cytokine expression in parental and aggressive H522M3 cells. An increase in IL-6, IL-8 and MCP-1 secretion was observed in aggressive H522M3 cells (Figure 6A), as assessed by densitometry of the cytokine blots (Figure 6B). Utilizing an ELISA, we further confirmed that secretion of these cytokines was increased by 2- to 5-fold in aggressive cells, compared to parental cells (Figure 6C). The significant elevated production of IL-6 and IL-8 was consistently observed in the two highly aggressive cells compared to parental cells with a prominent upregulation of MCP-1 in the H522M3 cells.

To further figure out the signaling pathway leading to cytokines production, the IL-6 and IL-8 levels were determined by ELISA after treatment with multiple pathway inhibitors. As shown in Figure 6D and Supplementary Figure 2A, 2B, while inhibitors of AKT and STAT3 pathway only partly suppressed production of IL-6 and IL-8 in H522M3 cells, NF-κB inhibitor Bay11-7082 or combined inhibition of AKT and STAT3 pathways completely inhibited secretion of these cytokines. Additionally, recombinant IL-6 (rIL-6), but not rIL-8 enhanced the production of both cytokines (Figure 6D) by activating AKT, STAT3 and NF-κB pathways (Figure 6E), indicating that an IL-6/NF-κB inflammatory loop is activated in aggressive NSCLC cells as illustrated in Figure 6F.

### miR-124 is down-regulated by NF-κB

**Figure 7:**
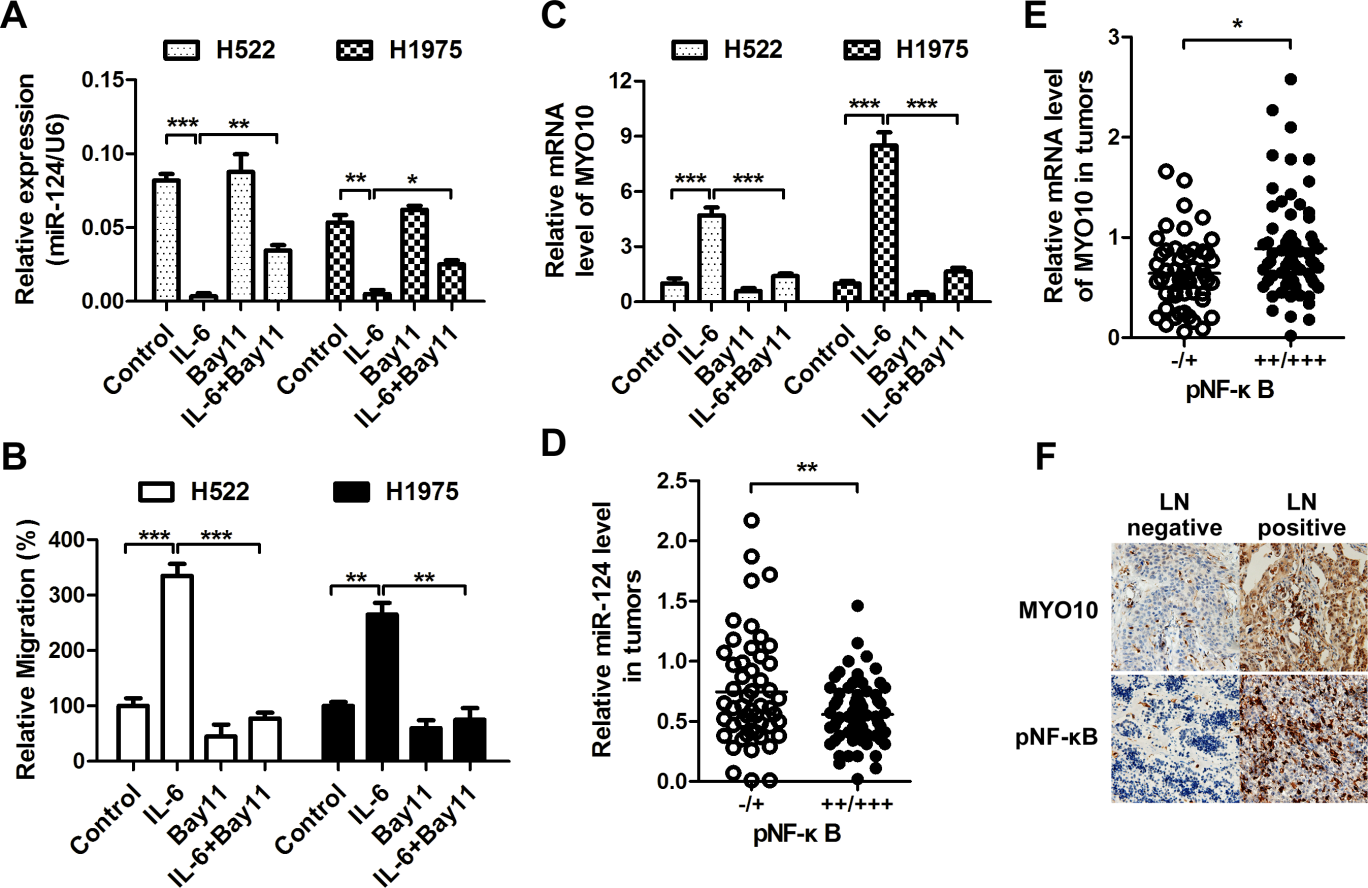
miR-124 is down-regulated by NF-κB **(A)** Cells were treated with rIL-6 (50 ng/ml), NF-κB inhibitor (10 μM Bay11-7082) or their combination, the expression of miR-124 was detected by qRT-PCR, **(B)** and the migratory properties were examined by transwell migration assays. *p < 0.05, **p < 0.01 and ***p < 0.001. **(C)** The MYO10 expression was also examined after rIL-6 or Bay11-7082 treatment. ***p < 0.001. (D and E) Correlative analysis of the **(D)** miR-124, **(E)** MYO10 mRNA, and pNF-κB in human NSCLC tumors (*n* = 20). pNF-κB was detected by immunohistochemistry (IHC), while expression of miR-124 and MYO10 was determined by qRTPCR. *p < 0.05, **p < 0.01. **(F)** Representative IHC images of MYO10 and pNF-κB in human NSCLC tumors with lymph nodes positive or negative.

The NF-κB transcription factor is known to control the expression of a number of miRNAs. We further determined whether the expression of miR-124 was regulated by the NF-κB-centered inflammatory loop. While IL-6 stimulation dramatically inhibited the expression of miR-124 (Figure 7A), an enhanced cell migration was observed in NSCLC cells (Figure 7B). Addition of NF-κB inhibitor Bay11-7082 greatly rescued the IL-6-mediated suppression of miR-124. The IL-6 induced cell migration was also suppressed after NF-κB inhibition. We also found an increase of MYO10 together with the activation of NF-κB in highly aggressive cells, and inhibition of NF-κB pathway by Bay11-7082 could markedly decrease the expression of MYO10 (Supplementary Figure 3A, 3B), suggesting the relation between MYO10 and NF-κB. We further determined the MYO10 levels under IL-6 stimulation, and found that MYO10 was obviously induced by recombinant IL-6, and was attenuated by addition of NF-κB inhibitor (Figure 7C). In addition, high NF-κB activity in human NSCLC tumors was significantly related to low miR-124 level (Figure 7D) and high MYO10 expression (Figure 7E and 7F). These results indicated the regulation of miR-124 and MYO10 by NF-κB.

## DISCUSSION

Increasing evidence demonstrated that the abnormal miRNA expression was detected in many human cancers, including non-small cell lung cancer (NSCLC), demonstrating various functions by interaction with tumor-related genes [21–22]. Now it is well established that some microRNAs may function as tumor suppressors or oncogenes regulating cell growth and tumorigenesis [23]. Recently, the role of miRNAs in tumor invasion and metastasis has been suggested in several cancer types [24–25]. However, only a few studies directly explored the relationship between miRNA changes and metastatic features in NSCLC.

Emerging evidence indicates that miR-124 was downregulated and inversely correlated with malignant tumor progression and poor prognosis [14, 26]. Recently, suppressive effects of miR-124 on tumor metastasis were reported in several cancers [15–17]. In this study, the potential involvement of miR-124 in NSCLC metastasis was investigated. Our results showed that miR-124 was deregulated in highly aggressive sub-cell lines and clinical node-positive specimens (Figure 2). The ectopic expression of miR-124 markedly suppressed the migratory and invasive capacities of NSCLC cells *in vitro* (Figure 3), suggesting the negative regulator of miR-124 in NSCLC invasion and metastasis.

MYO10 was further identified as a new direct and functional target of miR-124 using several different miRNA target prediction programs and experimental validation. MYO10 was recently reported to be essential for many cell processes, including wound healing, invadopodia formation and angiogenesis [27–29]. However, the role of MYO10 in NSCLC invasive growth and the gene expression regulation are both poorly understood. Recent observations elucidated that elevated expression of MYO10 contributes to aggressiveness and metastasis in breast cancer [30]. We found that the expression of MYO10 was commonly elevated in both aggressive sub-cell lines compared with their parental cells, further analysis demonstrated a significant high levels of MYO10 in human NSCLC tissues with lymphatic metastasis (Figure 4), implying the potential association of MYO10 with NSCLC metastasis. We further found a significant inverse correlation between the levels of MYO10 and miR-124, and MYO10 expression was remarkably suppressed by miR-124 over-expression, suggesting the inversely regulation of MYO10 by miR-124. A dualluciferase assay further confirmed that miR-124directly targeted MYO10 by binding to the 3′ untranslated region (3′-UTR) of MYO10 (Figure 4). While silence of MYO10 markedly impaired cell aggressiveness, forced expression of MYO10 reversed miR-124-mediated suppression of cell invasion, suggesting that miR-124 affected cell aggressiveness mainly by downregulating the expression of MYO10 proteins (Figure 5). Thus, the association between MYO10 and NSCLC aggressiveness and metastasis may make it a novel therapeutic target for NSCLC.

Cytokines and chemokines-molecular messengers involved in inflammation-have been shown to play an important role in tumor invasion and metastasis [31]. In this study, we also found an increase in IL-6 and IL-8 levels though NF-κB in highly invasive cell lines (Figure 6). IL-6 rather than IL-8 could activate the NF-κB pathway, which, in turn, trigger further IL-6 production forming a positive feedback loop. Previous studies have shown that miRNAs can be regulated at the transcriptional level or downstream of signaling pathways and thereby contribute to metastatic processes. NF-κB is a key transcriptional regulator linking tumorigenesis and inflammation [32]. Research increasingly suggests the influence of NF-κB in regulation of miRNAs [33]. In the present studies, we demonstrated that the IL-6-mediated activation of NF-κB pathway could suppress the expression of miR-124 promoting MYO10 expression and subsequent cell aggressiveness (Figure 7). It has been reported that IL-6-mediated STAT3 activation could suppress the expression of miR-124 by down-regulation of HNF4α, which could increase miR-124 expression by binding strongly to the promoter [34]. Since our data also revealed the activation of IL-6 inflammatory feedback loop via a STAT3-NF-κB pathway, we speculated that NF-κB may down-regulate miR-124 though repressing HNF4α expression in the metastatic lung cancer cells, needing further study.

In conclusion, our results proposed a novel regulatory mechanism driving NSCLC metastasis fostered by deregulation of miR-124 comprising of IL-6/NF-κB upstream inhibitory signal and MYO10 downstream executioners, providing an opportunity to develop new effective clinical therapies for NSCLC.

## MATERIALS AND METHODS

### Tissue specimens and cell lines

The NSCLC cell lines H522, H1975, H1299 and Calu-3 were obtained from the American Type Culture Collection (ATCC, Manassas, VA, USA). Highly aggressive sub-cell lines of H522 and H1975 were established using an *in vivo* selection systems as previously described [19]. All cells were cultured in RPMI 1640 medium (HyClone, Logan, UT) with 10% FBS and 100 units/mL of penicillin and 100 μg/mL of streptomycin (Invitrogen, Carlsbad, CA). The collection of tumor tissue from NSCLC patients were approved by our Institutional Review Board (IRB) and all tissue studied were provided by the Tissue Repository of the National Cancer Centre Singapore (NCCS).

### Microarray analysis and qRT-PCR assays

MiRNA was extracted from human NSCLC tissues and cells using mirVana miRNA Isolation Kit (Ambion, Carlsbad, CA, USA). The TaqMan microRNA assay and TaqMan universal PCR master (Applied Biosystems, Carlsbad, CA, USA) mix were used to examine the expression of miRNAs, and normalized by U6 small nuclear RNA. Profiling of miRNA was performed using miScript miRNA PCR Array (obtained from Qiagen in Valencia, CA), and the data was analyzed online (http://pcrdataanalysis.sabiosciences.com/mirna/arrayanalysis.php). Total RNA was extracted using TRIzol reagent (Invitrogen). First-strand cDNA was synthesized using Maxima First Strand cDNA Synthesis Kit (Thermo Scientific, Rockford, IL, USA). The gene expression was validated by real-time PCR using GoTaq qPCR Master Mix with SYBR green (Promega) with GAPDH as an internal control. The sequences of the sense (S) and antisense (AS) primers used in SYBR green quantitative PCR were as follows: MYO10-S: 5′-AAGTGGGGCAGGTAAAACCG-3′, AS: 5′-GCTCGTTCAACACAGGATGTC-3′; GAPDH-S: 5′-A ACAGCGACACCCACTCCTC-3′ and AS: 5′-CATACC AGGAAATGAGCTTGACAA-3′.

### Migration and invasion assays

Cell migration and invasion assays were performed using transwell chamber with 8 mm membrane pores (Corning, New York, NY, USA). For migration, 2 × 10^4^ transfected cells in serum-free medium were placed into the upper chamber per well with the non-coated membrane. For invasion, 1 × 10^5^ cells in serum-free medium were seeded into the upper chamber, which was pre-coated with Matrigel (BD, Bedford, MA, USA). After incubation for 24 h, the membrane inserts were removed from the plate, and non-invading cells were removed from the upper surface of the membrane using a cotton-tipped swab. The invaded cells on the underside were fixed in 100% methanol and stained with 0.1% crystal violet. Cells on the lower surface were counted and photographed under an inverted light microscope (Olympus, Tokyo, Japan). The assays were conducted three independent times.

### Wound-healing assays

For cell motility assay, cells were seeded in six-well plates to near confluence. A linear wound was carefully made by a 10 μL sterile pipette tip across the confluent cell monolayer, and the cell debris was removed by washing with phosphate-buffered saline (PBS) and incubated with RPMI 1640 medium (1% fetal bovine serum). The wounded monolayers were then photographed 48 h after wounding.

### *In vivo* metastasis assay

The *in vivo* metastatic potential of cells was analyzed in chicken embryos as described previously [20]. Briefly, H522M3 and H1975M3 cells transfected with P-miR-124 were inoculated on top of the chorioallantoic membrane of chicken embryos. Puregene DNA purification kit (Gentra, Minneapolis, MN, USA) was used to isolate the genomic DNA from lungs and livers. Metastasized cells to these organs were analyzed by quantifying primate-specific Alu sequences by qRT-PCR.

For the lung carcinoma metastasis model, 1.5 × 10^6^ cells carrying EGFP-tagged vectors in100 μL of PBS were injected into the tail vein of 6-week-old female BALB/c nude mice on day 0. After 3 weeks, the lungs were harvested and examined for EGFP-positive cells.

### Western blot

The whole-cell protein lysates were isolated using RIPA and protein concentrations were determined using the Bradford protein assay (Bio-Rad, Hercules, CA, USA). Antibodies for Western blot were: phospho-STAT3 (pSTAT3, Tyr705), phospho-AKT (pAKT, Ser473), phospho-ERK (pERK1/2, Thr202/Tyr204) and GAPDH from Cell Signaling Technology (Denvers, MA, USA); phospho-NF-kB p65 (pNF-κB, Ser536) and MYO10 (ab189259) from Abcam (Cambridge, MA, USA).

### Transfection

Stable H522M3/pRTR-miR-124 and H1975M3/pRTR-miR-124 cells were generated by transfection of the episomal expression vector pRTR using Fugene6 (Roche). The cells were then selected in the presence of 3.0 μg/mL puromycin (Sigma) and validated by flow cytometry for GFP-positive cells 72 h after addition of doxycycline (Sigma). pre-miRNAs (P-miRNAs), anti-miRNA, siRNAs and respective negative controls (Applied Biosystems, Foster City, CA, USA) were transfected into cells using Lipofectamine 2000 Reagent (Invitrogen) according to the manufacturer's protocol.

### Luciferase reporter assay

3 × 10^4^ cells were seeded into 12-well plates. After 12 h, 200 ng of P-miR-124 plasmid was co-transfected with 50 ng of pGL3-MYO10-3′-UTR. Cells were lysed 36 h after transfection, and luciferase activity was measured using a Dual-Luciferase system (Promega, Madison, Wisconsin) according to the manufacturer's protocol. Mutations in the miR-124 seed-matching sequences were made with the QuikChange Mutagenesis Kit (Stragene, La Jolla, Calif., USA).

### Human cytokine antibody array and ELISA

Conditioned media was collected from H522 and H522M3 cells after cultured for 24 h. Relative amounts of cytokine levels were analyzed by the Human Cytokine Array kit (Panel A, ARY005, R&D Systems) according to the manufacturer's instructions. For ELISA assay, the conditioned medium collected from cells was used for detection of cytokine expression pattern using Human IL-5/IL-6/IL-8/MCP-1 ELISA Kit (BioLegend, CA, USA). For some experiments, cells were treated by MEK1/2 (U0126), p38 kinase (SB203580), JNK (SP600125), STAT3 (Stattic) and PI3K/AKT (LY294002), NF-κB (Bay11-7082) inhibitors (all from Selleck Chemicals, Houston, TX, USA), or recombinant human IL-6/IL-8 (R&D Systems, Minneapolis, MN, USA) for 24 h, and the medium was collected for ELISA analysis.

### Immunohistochemistry (IHC)

Tumors were fixed in formalin and embedded in paraffin. 4 μm-thick sections were made and incubated with 3% hydrogen peroxide to quench endogenous peroxidase activities. Antigen retrieval was performed by heat mediation in citrate buffer (pH 6) (Dako). The slides were blocked with 10% goat serum before incubating with primary antibody. Then, the samples were incubated with the antibodies against phospho-NF-κB p65 (pNF-κB, Abcam) and MYO10 (Abcam) overnight in a humidified container at 4°C. PBS without primary antibodies was applied as the negative control. Immunohistochemical staining was performed with the 3-3′-diaminobenzidine (DAB) and were counterstained with hematoxylin. The immunohistochemistry results were scored and classified into two groups according to the average staining intensity and area.

### Statistical analysis

Data are expressed as mean ± s.e.m.. The significance of differences between groups was assessed by *t*-test or one-way ANOVA. All analyses were performed with GraphPad Prism program version 5 (GraphPad Software, USA). Two-tailed tests were used, and the asterisk indicates a statistically significant difference: “*” means *p* < 0.05, “**” means *p* < 0.01, “***” means *p* < 0.001.

## SUPPLEMENTAL FIGURES AND TABLES


