# Supplementary material for: NF-κB-mediated miR-124 suppresses metastasis of non-small-cell lung cancer by targeting MYO10
Source: Oncotarget. 2015 Jan 29;6(10):8244–54. doi: 10.18632/oncotarget.3135 (PMC4480748; doi:10.18632/oncotarget.3135)
Supplement: Supplementary file 1 [file oncotarget-06-8244-s001.pdf]

SUPPLEMENTAL FIGURES AND TABLES

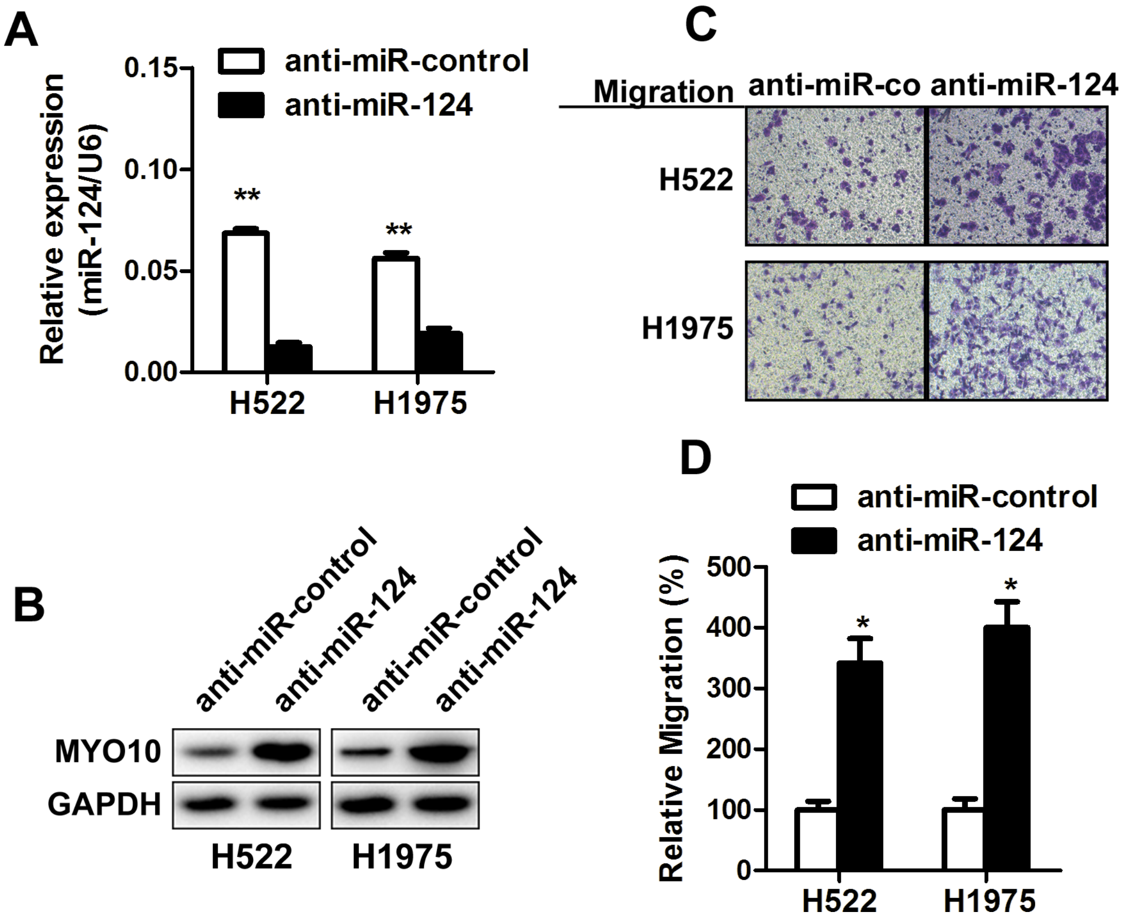

Supplementary Figure 1: Silencing of miR-124 in the NSCLC cells. Anti-miR-124 was transfected into H522 and H1975 cells. **(A)** Expression of miR-124 was examined by qRT-PCR and significant silencing of miR-124 in both cells was demonstrated. $**p < 0.01$. **(B)** The expression of MYO10 after transfection was detected by Western blot. **(C)** Representative IHC images of cell migration were showed, and **(D)** the migrated cells were summarized in the bar graph. $*p < 0.05$.

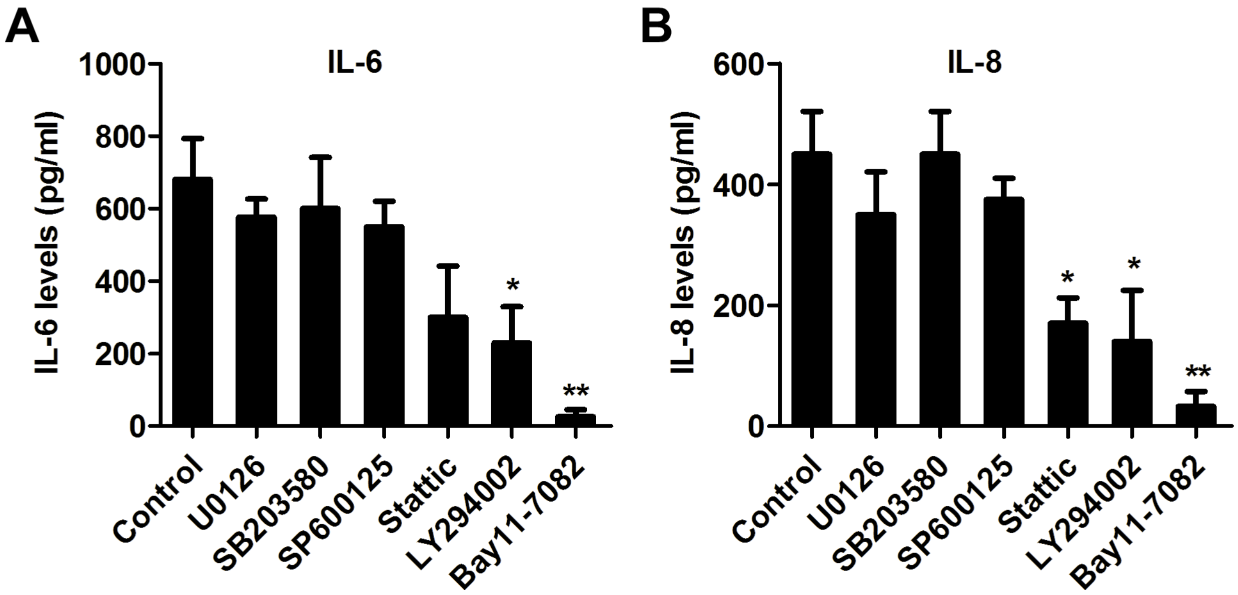

Supplementary Figure 2: Effects of multiple pathway inhibition on the production of IL-6 and IL-8. H522 cells were treated with MEK1/2 (U0126, 20 µM), p38 kinase (SB203580, 20 µM), JNK (SP600125, 50 µM), STAT3 (Stattic, 5 µM) and PI3K/AKT (LY294002, 10 µM), NF-κB (Bay11-7082, 10 µM) inhibitors, and the **(A)** IL-6 and **(B)** IL-8 levels were examined by ELISA. $*p < 0.05$, $**p < 0.01$.

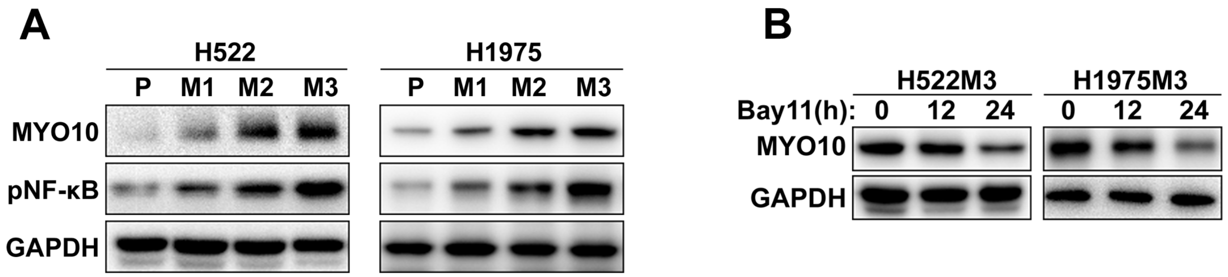

Supplementary Figure 3: The regulation of MYO10 by NF-κB pathway. **(A)** Western blot analysis the expression of MYO10 and pNF-κB in parental and aggressive sub-cell lines. **(B)** H522M3 and H1975M3 cells were treated with 10 µM Bay11-7082 for indicated time and the MYO10 expression were detected.

Supplementary Table S1: Analysis of the fold regulation in the microRNAs (miRNAs)

H522-M3/P			H1975-M3/P		
Position	Mature ID	Fold Regulation	Position	Mature ID	Fold Regulation
D10	hsa-miR-124-3p	−131.8522	D10	hsa-miR-124-3p	−176.2729
A12	hsa-let-7g-5p	−95.4128	E07	hsa-miR-30a-5p	−51.3277
C03	hsa-miR-15b-5p	−53.3018	C09	hsa-miR-125b-5p	−33.5522
B09	hsa-miR-181b-5p	−42.4035	E12	hsa-miR-30d-5p	−23.5067
B01	hsa-miR-30c-5p	−38.8393	A12	hsa-let-7g-5p	−20.3224
D01	hsa-miR-125a-5p	−33.1157	B08	hsa-miR-425-5p	−15.9078
B03	hsa-miR-185-5p	−29.8456	E05	hsa-miR-144-3p	−14.1069
B11	hsa-miR-30b-5p	−26.8983	G02	hsa-let-7c-5p	−13.0412
G08	hsa-miR-20a-5p	−24.2982	E11	hsa-miR-143-3p	−12.8023
A02	hsa-miR-9-5p	−22.8815	E01	hsa-miR-25-3p	−12.7433
C05	hsa-miR-194-5p	55.0965	F08	hsa-miR-93-5p	−10.8403
A10	hsa-miR-32-5p	28.3883	B01	hsa-miR-30c-5p	14.7741
D08	hsa-miR-423-5p	22.3761	C10	hsa-miR-99a-5p	14.1723
C06	hsa-miR-210-3p	17.8835	A10	hsa-miR-32-5p	12.0281
A03	hsa-miR-150-5p	16.6089	C03	hsa-miR-15b-5p	10.6172

NOTE: Fold-Change (2^(−Delta Delta Ct)) is the normalized gene expression (2^(−Delta Ct)) in the third round cells (M3) divided the normalized gene expression (2^(−Delta Ct)) in the parental cells (P).

Supplementary Table S2: Predicted targets of miR-124

Symbol	A	B	C	D	E	F	G	H	I	J	K
C10orf56	0	1	1	1	0	0	1	1	1	1	1
MYO10	0	1	1	1	0	0	1	1	1	1	1
PLDN	0	1	1	1	0	0	1	1	1	1	1
LRRC1	0	1	1	1	0	0	1	1	1	1	1
TMEM109	0	1	1	1	0	0	1	1	1	1	1
PTBP1	0	1	0	1	0	1	1	1	1	1	1
CTDSP2	0	1	1	0	0	1	1	1	1	1	1
B4GALT1	0	1	1	1	0	1	1	1	0	1	1
AHR	0	1	1	1	0	1	1	1	0	1	1
SLC16A1	0	1	1	1	0	1	1	1	0	1	1
PTTG1IP	0	0	1	1	0	1	1	1	1	1	1
SERP1	0	0	1	1	0	1	1	1	1	1	1
CHODL	0	0	1	1	0	1	1	1	1	1	1

Note: A: Diana, B: Microinspector, C: Miranda, D: Mirtarget2, E: Mitarget, F: Nbmirtar, G: Pictar, H: Pita, I: Rna22, J: Rnahybrid, K: Targetscan. 1 means “predicted” and 0 means “not predicted”.
